# Cough Hypersensitivity Syndrome: Why Its Use Is Inappropriate in Children

**DOI:** 10.3390/jcm12154879

**Published:** 2023-07-25

**Authors:** Anne B. Chang, Richard S. Irwin, Hannah E. O’Farrell, Peter V. Dicpinigaitis, Suhani Goel, Ahmad Kantar, Julie M. Marchant

**Affiliations:** 1 Australian Centre for Health Services Innovation, Queensland University of Technology, Brisbane, QLD 4059, Australia; 2 Department of Respiratory and Sleep Medicine, Queensland Children’s Hospital, Brisbane, QLD 4101, Australia; 3 NHMRC Centre for Research Excellence in Paediatric Bronchiectasis (AusBREATHE), Child Health Division, Menzies School of Health Research, Charles Darwin University, Darwin, NT 0810, Australia; 4 Division of Pulmonary, Allergy, and Critical Care Medicine, Department of Medicine, UMass Memorial Medical Center, Worcester, MA 01605, USA; 5 Division of Critical Care Medicine, Albert Einstein College of Medicine and Montefiore Medical Center, Bronx, NY 10461, USA; 6 Somerville House, South Brisbane, QLD 4101, Australia; 7 Pediatric Asthma and Cough Centre, Istituti Ospedalieri Bergamaschi, University and Research Hospitals, via Forlanini 15, Ponte San Pietro-Bergamo, 24036 Bergamo, Italy

**Keywords:** cough, children, review, hypersensitivity, evidence

## Abstract

In children and adults, chronic cough is a common symptom presenting to health professionals worldwide. It is internationally accepted that children with chronic cough should be managed with pediatric specific management guidelines. The newly proposed clinical entity of ‘cough hypersensitivity syndrome’ has gained significant attention in adult literature. Given the significant differences between childhood and adult chronic cough, including in respiratory physiology and anatomy, and cough sensitivity, we address the suitability of the use of cough hypersensitivity syndrome in children. We explore these differences between childhood and adult chronic cough, explain what cough hypersensitivity is and highlight why the term cough hypersensitivity syndrome should not be used in children.

## 1. Introduction

Globally, coughs of varying durations are the most common symptom leading to medical consultation [1]. Clinicians need to be cognizant that the vast majority of pediatric acute coughs are benign (i.e., resolve spontaneously). However, cough, especially when chronic, is bothersome to both parents and children, has a high healthcare burden and impairs their quality of life (QoL) [2,3,4]. Additionally, the cough may represent the first presentation of an underlying lung disease and may progress to chronic cough in a subset of children [5,6]. Its importance is often unrecognized by doctors.

In the late 1990s to early 2000s, guidelines recommended that children with chronic cough be managed in the same manner as adults [7] and even suggested large doses of oral corticosteroids as a treatment trial [8]. With the increased availability of robust clinical research data, including randomized controlled trials (RCTs) [9,10], it is now widely accepted that pediatric-specific approaches should be used to achieve the best clinical outcomes [11]. Thus, there is a need to focus on pediatric chronic cough in light of the push by some in the field to convert undifferentiated etiologies of chronic cough into the umbrella of ‘cough hypersensitivity syndrome’.

## 2. Defining Chronic Cough in Children

Pediatric (children aged < 14 years) chronic cough, unlike in adults, is defined as a daily cough lasting for >4 weeks [11]. In adults, chronic cough is defined as a daily cough lasting for >8 weeks [12]. There are several reasons for the shorter duration used in children. The most important ones are that (i) this duration is based on the natural history of viral-related cough and (ii) to ensure that a serious underlying condition is not missed [11,13]. A multicenter study that used a pediatric cough algorithm found a serious potentially progressive underlying respiratory illness (bronchiectasis, aspiration lung disease or cystic fibrosis) in 18% of 346 children [2]. Additionally, published studies that systematically assessed the outcomes of individual children at a children’s specialist hospital who had acute cough that persisted for >4 weeks found a new and serious chronic lung disease (e.g., chronic pneumonia, bronchiectasis) in up to 30.8% of children [5,6]. In addition to age-related immunological reasons, the high contact exposure of children to viral infection due to close contacts in daycare and schools is associated with the higher frequency of viral infections in children (compared with adults) [14].

## 3. What Is Cough Hypersensitivity Syndrome

Cough hypersensitivity represents “increased neural responsivity to a range of stimuli affecting the airways and lungs and other tissues innervated by common nerve supplies” [15]. Clinically it presents as “excessive coughing often in response to relatively innocuous stimuli” [15] and includes allotussia, which is a cough triggered by nontussive stimuli such as smells or the eating of biscuits [16]. Cough sensitivity is measured by the concentration of the cough stimulant that triggers the number of coughs, e.g., C2 represents a concentration that stimulates two or more coughs and C5 represents a concentration that stimulates five or more coughs [17]. There are various cough stimulating-agents including citric acid [18], acetic acid [19], distilled water or fog [20] and capsaicin [17], which is the most widely used method. However, there is currently no defining cut-off that determines cough hypersensitivity.

Early studies in adults described increased cough sensitivity in those with an upper respiratory tract infection (compared with controls) [18] and the temporal relationship with increased sensitivity of the cough reflex in those with a cough related to treatment with angiotensin-converting enzyme inhibitors [21]. Following these earlier studies, subsequent studies in both children [22] and adults [23] have demonstrated that cough sensitivity decreases with successful treatment of the cough. However, in adults, cough can be unrelenting when their primary underlying etiology cannot be identified, such as in the cases of refractory chronic cough (RCC) and unexplained chronic cough (UCC) [24]. Chronic cough and cough hypersensitivity is more prevalent in adult women, and this is said to be reflected in the lower threshold to elicit cough with inhaled capsaicin [16]. These findings have been found in both healthy adult women and those with chronic cough, as summarized in a recent review [16].

The definition of ‘cough hypersensitivity syndrome’, although important, seems unclear to some even in the adult literature. For example, some adult-focused groups have promoted cough hypersensitivity as a syndrome that “represents a clinical entity in which chronic cough is a major presenting problem, regardless of the underlying condition” [25]. As such, all causes of chronic cough represent cough hypersensitivity syndrome. In contrast, others logically emphasize that chronic hypersensitivity syndrome should only be inferred when the cough is refractory to all treatments or unexplained after an evidence-based workup has been faithfully followed. Their reasoning is that observing abnormal sensations in the pharynx or larynx, (e.g., tickle or itch, or an uncontrollable urge to cough) has been of limited value in predicting outcomes when treating a variety of causes of cough. Likewise, obtaining tests that reflect an overactive cough reflex is also of limited value in predicting outcomes when treating a variety of causes of cough. Such observations have been exemplified in a study of Mai et al. [26] where most patients with these abnormal sensations recuperated through a specific treatment for the conditions uncovered by typical diagnostic methods. While we recognize that cough hypersensitivity syndrome is at the root of a truly refractory or unexplained chronic cough in adults, most patients referred to one of this paper’s adult-based authors in regard to this syndrome did not have faithful workup to an evidence-based diagnostic and intervention protocol, and were found to have other causes when such protocols were faithfully followed [27]. Given the variable definition, we refer readers to papers on this topic for various adult descriptions [25,26] of this syndrome. With its variable definition, cough hypersensitivity can be a stand-alone entity or can co-exist in conjunction with other cough etiologies or associations, e.g., asthma, gastro-esophageal reflux, or Arnold’s ear reflex [28,29,30].

## 4. Why Assigning Cough Hypersensitivity Syndrome as a Diagnostic Concept Is Inappropriate in Children

The common underlying etiologies of chronic cough in children are different to those in adults and thus evidence-based guidelines for adults [12,27] and children [11,31] differ in countries such as the USA. These common etiologies of chronic cough in children are dependent on the setting (country, type of clinic, sampling frame, etc.) and have been summarized in a systematic review [32]. Of these studies, the largest was an Australian multicenter study where 346 newly referred children with chronic cough were systematically managed in accordance with the American College of Chest Physicians’ guidelines [2]. In that study, the frequency of etiologies was significantly different in dissimilar settings (*p* = 0.0001), with 17.6% of children having a serious underlying diagnosis (bronchiectasis, aspiration, or cystic fibrosis). Overall, the most common etiology was protracted bacterial bronchitis [2]. The systematic evaluation of children with chronic cough has contrasting features to that of adults; for example, in the duration of cough (>4 weeks in children versus >8 weeks in adults [11,12,27]) and in the assessment of the likelihood of underlying features based on specific pointers [11,33], including wet cough [34].

The use of cough meters to objectively measure cough frequency and cough sensitivity as outcome measures within a clinical trial was undertaken in children [35] prior to any such studies in adults. Indeed, the importance of controlling inspiratory flow in the assessment of cough sensitivity tests was first understood in children [17] prior to adult data. Although heightened cough receptor sensitivity in some conditions has been known in children for decades [19,36,37,38], the concept of cough hypersensitivity as an overarching approach and/or syndrome for all causes of chronic cough, promoted in adults by some groups [25], should not be used in children for several important clinical reasons. These reasons are summarized below.

Firstly, in children, specific diagnosis aids in the appropriate management and subsequent resolution of the chronic cough [33]. Thus, pediatricians diagnose specific disease entities even when the underlying pathology may include a temporal hypersensitive state or response. Likewise, eczema in children is not called skin hypersensitivity nor is asthma-related wheeze referred to as airway hypersensitivity, although both are clear examples of hypersensitivity conditions.

Secondly, in children, cough is a symptom of many underlying diseases and not a disease itself. Referring to all these various underlying diseases using a single umbrella term (i.e., cough hypersensitivity syndrome) risks inappropriate treatment based on treating the symptom of cough rather than using the etiology-based approach. The etiology-based approach—treatment based on managing the underlying disease results in resolution of the chronic cough and as recommended by the American College of Chest Physicians’ chronic cough guideline [11]—has been repeatedly proven to be effective in children with chronic cough. This has been shown in multiple cohort studies (as described in a systematic review [39]) and in two multicenter RCTs [9,10].

Thirdly, there are key major physiological differences between children and adults [40] that result in different chronic cough-related phenotypic expressions [41]. These major physiological differences include cough hypersensitivity, mentioned above [42]. Conversely, age influences cough sensitivity in children [42] but plays little role in adults. Other aspects of this are summarized in the section below.

Further, in children (unlike adults), persistent cough sensitivity has not been found in the common causes e.g., viral infections in children with asthma [22], to significantly change post intervention e.g., post adenoidectomy [43], or in situations where cough is expected to occur (post-exercise in children with asthma [44]). Additionally, cough sensitivity has been shown to be not consistently elevated in children, e.g., C5 (concentration of capsaicin that trigger five or more coughs) was no different between children with asthma and controls (though C2 was significantly different) [45], whereas the reverse was described in children with cystic fibrosis [37].

Cough hypersensitivity is akin to pain hypersensitivity, postulated in the 1990s [38], and has recently been shown to share a common pathophysiology e.g., the role of neurotrophins [46]. Pain hypersensitivity is age-dependent and rare in children but becomes more common in adolescents [47]. Cough hypersensitivity is a key feature of refractory chronic cough and/or idiopathic/unexplained cough in adults (and can be ameliorated by medications that are effective in neuropathic pain, e.g., established medications, gabapentin, or amitriptyline [15]) but this is not the case in children where it is not a recognized entity.

Further, the prevalence of Arnold’s ear–cough reflex in children with chronic cough is similar to that in healthy children [28]. This is in contrast with adults, where the prevalence of the reflex is 11-fold higher in those with chronic cough compared with healthy adults and adults with respiratory disease without cough [28]. However, children with chronic cough had a similar prevalence of Arnold’s nerve reflex as did healthy children (2%) [28]. This suggests that chronic cough/refractory cough in adults is likely due to a significant extent to a hypersensitive cough reflex being caused by triggers that generally do not cause cough in adults without chronic cough (e.g., eating biscuits, smells, etc.). In contrast, chronic cough in children is most likely related to the continuous stimulation of a normal cough reflex by persistent triggers, such as airway mucus, infection, inflammation, viral/post-viral inflammation, etc. [48]. These data led the same authors to conclude that cough hypersensitivity syndrome is an acquired condition in adults [48].

Of all the etiologies of cough, asthma has been studied the most, as has its relationship with cough sensitivity. The relationships of cough sensitivity and other markers of the severity of asthma in both acute and non-acute asthma in children are summarized in Table 1 [29]. These studies have found that cough hypersensitivity in children with asthma has a poor relationship with other asthma markers (e.g., eosinophilic cationic protein, interleukin-8, sputum eosinophils and airway hyperresponsiveness (AHR)). Additionally, Japanese data suggest that cough and AHR are independent pathways [49,50]. In contrast, in adults with cough and asthma, there is an emphasis on cough hypersensitivity with laryngeal paraesthesia, allotussia and hypertussia [29]. Thus, it is unsurprising that the approach to children with asthma and cough is different to that in adults. The emphasis on the definition and treatment of the underlying etiologies is key in children [11], whereas neuromodulators (e.g., gabapentin) are considered for the treatment of adults with asthma and cough [29].

## 5. Other Major Differences between Children and Adults with Respect to Cough-Related Issues

Children and adults share some similarities in the physiology of the respiratory system. However, there are also distinct differences between children and adults. These have been summarized in a recent review [16].

The young child’s respiratory physiology and anatomy differs substantially from that of an adult. These differences encompass respiratory muscle development and chest and airway compliance. The effects of these on chronic cough include a lack of the ability to expectorate excessive lower airway secretions such that the term ‘wet cough’ is used in children instead of productive cough [34]. Differences in bronchial and alveolar development, e.g., reduced pores of Kohn in children (in addition to shape and angle of right middle lobe take-off) also exist, resulting in conditions that present as chronic cough-like right middle lobe syndrome, another entity in children [53]. The key factors with respect to cough include plasticity or the adaptability of the cough reflex, which are known to be age-related in animals [54], and it is also likely that age-related maturation occurs in the human cough reflex [55]. Of particular importance in cough sensitivity are the sex differences, which are pronounced in adults but absent in prepubertal children [37,42,56]. Thus, the propensity of chronic cough to be more common in adult females [16] is not present in prepubertal female children. Cough sensitivity in children is instead influenced by airway caliber (forced expiratory volume in 1 s, FEV1) and age [37]. Recent data in children with asthma have shown that changes in gene expression differ between pre- and post-menarcheal females i.e., that there is a shift from a predominantly innate to an adaptive immunity [57]. Authors demonstrating this evidence have suggested that “gene expression changes associated with asthma and may explain sex differences in prevalence” [57]. It is likely but remains unknown if similar changes related to cough sensitivity are also present.

The underlying physiology of children results in some important differences in their metabolism and response to medications when compared with adults [58]. Specific examples of these differences include differential recommendations or the use of opiates (contraindicated in children but has a role in adults) and the frequency of cough as an adverse effect of angiotensin-converting enzyme inhibitors (less common in children compared with adults) [11].

Other key differences include the ontology of the immune system that influences infection rates and inflammatory responses to infections. The young child’s immune development is pliable and prone to epigenetic influences [59]. In children, >90% of respiratory viral infections resolve within three weeks, while post-infectious cough in adults lasts longer [60].

These differences likely account for the different common etiologies of chronic cough in children [32], which are different than the common etiologies in adults, and the need for pediatric-specific algorithms. Other study-related differences include measurement outcomes e.g., cough-specific quality of life (QoL) tools [61] where adult cough outcome measures include issues such as urinary incontinence, which is not relevant to children [62].

## 6. Summary

It is now widely accepted that childhood chronic cough is different to that of adults and international guidelines recommend that pediatric-specific approaches should be used. These pediatric approaches include key fundamental differences such as the definition of chronic cough (>4 weeks in children), pediatric-specific diagnostic algorithms and, indeed, different common etiologies. Key differences in the physiology and anatomy of the respiratory system, as well as an immature immune system and plasticity of the cough reflex during childhood are explanatory as to why these differences exist. Importantly, investigation of childhood chronic cough with appropriate diagnostic algorithms, including RCT data, has repeatedly shown that, after a specific diagnosis is made and treatment has begun, there is a resolution to the cough.

Whilst children may have heightened cough receptor sensitivity in some conditions, its treatment leads to resolution of the cough, irrespective of a hypersensitive state which resolves with the resolution of the cough. Labeling a child with the term ‘cough hypersensitivity’, when dealing with chronic cough is likely to be misleading and possibly harmful if the underlying cause is not sought and managed. Cough hypersensitivity in pediatric asthma has been studied and has not been shown to be linked to other indicators of asthma severity. Further, unlike in adults, persistent cough hypersensitivity has not been found in pediatric studies of common causes. Importantly, studies on Arnold’s ear reflex in children and adults have suggested that cough hypersensitivity syndrome is an acquired condition in adults.

In summary, the diagnostic entity of cough hypersensitivity syndrome that is promoted in adults is inappropriate in children and should not be used.

## Figures and Tables

**Table 1:** Studies that have examined cough sensitivity in children with asthma (Table, completed by AC, was reproduced from Lancet Respir Med [29] with permission).

First Author Year, Country	Study Design	Inclusion and Exclusion Criteria	Asthma Parameters Used	Cough Measurement	**N; Age;** Follow-Up Duration	Main Aim(s)	Main Study Findings	Implication/Conclusion
Chang [22] 1997, Australia	Prospective longitudinal	Inclusion: Children > 6 yrs hospitalised with asthma Exclusion: Concurrent other chronic disease	Acute asthma severity: functional severity score (FSS) and acute asthma severity (AAS) scale. Coughers: always or usually cough with asthma symptoms. Non-coughers: Sometimes/with infections only or never cough	CRS using capsaicin (C2 and C5)	Coughers *n* = 15; median age = 9 yrs (IQR 3.6) Non-coughers *n* = 16; 9 yrs (IQR 3.1) Tests undertaken when hopsitalised, repeated at 7–10 days and 4–6 W	Hypothesis: “in children with asthma who cough as a major symptom, CRS is heightened during an acute severe exacerbation of asthma but not in the non-acute phase and airway calibre or its change correlates with CRS”	CRS of “coughers” significantly higher than non-coughers (mean difference log C2 0.77 umol (95% CI 0.35 to 1.18), C5 0.72 (0.26 to 1.18)) during acute asthma but not after the exacerbation. FEV_1_ and its change correlated with neither CRS nor its change. Both groups similar in smoke exposure, asthma meds, FSS, AAS scores	“CRS is heightened in acute severe asthma in the subgroup of children who have cough as a significant symptom with their asthma episodes”
Chang [37] 1997, Australia	Cross section, single centre		Asthma: recurrent episodes of wheeze and tachypnoea that responded to salbutamol	CRS using capsaicin (C2 and C5)	Asthma *n* = 35, median age 10.3 yrs (range 6–16); recurrent dry cough *n* = 47, age 9 (5–17); CF *n* = 27, age 11.9 (7–18); controls *n* = 100, age 10.7 (6–17)	To determine if CRS is (1) altered in children with asthma, recurrent cough, and cystic fibrosis (CF) and (2) influenced by age, gender or FEV_1_	CRS increased in children with recurrent cough, and reduced in children with cystic fibrosis, compared with children with asthma and controls. Age influenced CRS in controls. In children with asthma, C2 and C5 influence by FEV_1_ % was predicted	Children should be matched for age and FEV_1_ when cough sensitivity is used in comparative studies
Chang [51] 1997, Australia	Cross section, single centre	Inclusion: children with asthma and healthy controls. Exclusion: not described	Non-acute asthma. Controls: healthy children	C5, AHR to hypertonic saline (HS), spirometry	Asthma *n* = 12, mean age = 10.3 yrs Controls *n* = 9, mean age = 12.7	To determine whether inhalation of capsaicin for the CRS test before HS challenge alters AHR of children with and without asthma	Both groups: capsaicin did not alter FEV_1_ Asthma: mean of the difference in log PD_15_ was within the equivalence range of the HS challenge in children with asthma.	CRS testing with capsaicin did not alter AHR to HS. HS-induced bronchoconstriction and capsaicin likely stimulate different pathways
Chang [52] 2002, Australia	Prospective cohort	Inclusion: baseline: stable asthma (no respiratory tract infection or exacerbation for at least 4 weeks) and retested during days 1, 3, 7, and 28 of an exacerbation. Exclusion: other chronic respiratory disease	Asthma in non-acute and acute exacerbation	Asthma diary, quality of life, lung function (FEV_1_, FEV_1_ variability), AHR to HS, cough diary, CRS, and inflammatory markers (sputum IL8, ECP and MPO; and serum ECP)	Baseline *n* = 21; exacerbation *n* = 11. Median age 10.5 yrs, IQR = 3.9	To examine and relate common asthma indices (QOL, AHR, lung function, asthma diary) with cough indices (CRS, cough diary) and markers of eosinophilic and neutrophilic inflammation (serum ECP, sputum ECP, IL-8 and MPO) in children with asthma during a non-acute, acute, and resolution phase of asthma	CRS outcome measures (C2 and C5) did not correlate with any marker of clinical severity (asthma score, cough score, QOL), pulmonary function indices (FEV1, forced vital capacity (FVC), FEV1 variability) or inflammatory marker (IL-8, serum ECP, sputum ECP, serum eosinophils, sputum eosinophils, sputum neutrophils, MPO) of asthma during any of the test days (baseline, D1, D3, D7, and D28).	No relationship between CRS to sputum eosinophils, IL8, ECP or MPO. CRS does not reflect eosinophilic airway inflammation.
Ferenc [44] 2018, Slovak Republic	Cross section, single centre	Inclusion: asthma, baseline FEV_1_ > 80% and no respiratory symptoms for at least 4 W. Exclusion: not described.	Asthma: “wheezing, cough, dyspnea or chest tightness at rest OR on exercise and a positive response to exercise challenge”	CRS using capsaicin (C2 and C5) Broncho-dilators withheld for 72 h	*n* = 42; mean age 14.1 yrs ± SD 2.1. No follow-up	“clarify changes of cough reflex sensitivity before and after exercise challenge testing in asthma children”	C2 pre-exercise challenge median = 9.77 um/L (95%CI 6.10–10.99); post = 7.32 (6.10–14.65) (*p* = 0.58). C5 respective values: pre = 19.53 (14.65–80.57) post = 39.06 (24.42–58.59) *p* = 0.09	Cough reflex not significantly altered post exercise. Asthma medications not described and whether children had cough with exercise not mentioned
Kunc [45] 2020, Slovak Republic	Cross section	Inclusion: asthma, baseline FEV_1_ > 80% and no respiratory symptoms for at least 4 W. Exclusion and controls: not described.	Asthma: “wheezing, cough, dyspnea or chest tightness at rest OR on exercise and a positive response to exercise challenge”	CRS using capsaicin (C2 and C5) Broncho-dilators withheld for 72 h	Asthma *n* = 25; mean age 9 yr SD 1; Controls *n* = 15; 8 ± 1 No follow-up	“clarify changes of cough reflex sensitivity in asthma children”	C2: Asthma group mean 4.25 µmol/L (95%CI 2.25–8.03) vs. control 10.61 (5.28–21.32) *p* = 0.024. C5: 100.27 (49.30–203.93) vs. 56.53 (19.69–162.35) respectively, *p* = 0.348	C2 increased in children with asthma but not C5. Inconsistent results. Asthma medications not described
Mochizuki [50] 1995, Japan	Cross section for aim 1, RCT for aim 2	Inclusion: Stable asthma and no upper respiratory tract infections for >2 W, no spontaneous coughing/other symptoms, no medication for >16 h before the tests	Stable asthma (recurrent dyspnoea with wheeze and diagnosed for >2 yrs).	PD_20_ of ultrasonically nebulized distilled water (UNDW) and histamine. Concentration of acetic acid (AA) inducing the first cough (cough threshold)	Study 1: *n* = 40. Mean age 11.2 yrs SD 2. Study 2: *n* = 12. Mean age 11.3 yrs 2.4 SD 2	Aims: to study (1) the relationship between UNDW and acetic acid inhalation challenge compared to histamine; and (2) the effect of inhaled furosemide	UNDW-PD_20_ correlated with AA cough threshold (r = 0.57, *p* < 0.001). Histamine-PC_20_ did not correlate with AA cough threshold or UNDW-PC_20_. “Frusemide exerted protective effect on UNDW and AA but not on histamine”.	Mechanism of hyper-responsiveness to UNDW and AA-induced cough may be similar but is dissimilar to histamine
Shimuzu [49] 2016, Japan	Cross sectional, Single centre	Inclusion: asthma without respiratory infections at >4 W and no asthma-related symptoms at the time of the study. No meds for >12 h before the tests. Exclusion: not mentioned.	Asthma: recurrent dyspnoea with wheeze, and diagnosis established for >1 yr	Histamine-induced FEV_1_ change, CRS to acetic acid (AA), Spirometry, AHR to histamine	*n* = 19, mean age 10·6 yr, standard error of mean 0·6	Determine: (1) effect of histamine-induced broncho-constriction and salbutamol-induced bronchodilatation on AA cough threshold”, and (2) relationship between AA cough threshold and AHR to histamine in children with asthma	No relationship between CRS thresholds and change in FEV_1_, and PD_20_	CRS to acetic acid and bronchomotor tone are independent pathways in children with asthma

AHR = airway hyperresponsiveness; AAS = acute asthma severity scale; C2 = amount of stimulant that stimulated at least 2 coughs; C5 = amount of stimulant that stimulated at least 5 coughs; CRS = cough receptor sensitivity; ECP = eosinophilic cationic protein; FEV_1_ = forced expiratory volume in one second; FSS = functional severity score; FVC = forced vital capacity; HS = hypertonic saline; IL8 = interleukin 8; IQR = interquartile range; MPO = myeloperoxidase; PD_15_ = provocative dose causing a 15% fall in FEV_1_; PD_20_ = provocative dose causing a 20% fall in FEV_1_; SD = standard deviation; W = week(s); yr = year(s).

## Data Availability

Not applicable.
